# Frequent Sweetened Beverage Consumption Is Associated With Accelerated Biological Aging: Evidence From a Population‐Based Study and Gut Microbiota Analysis

**DOI:** 10.1002/advs.76460

**Published:** 2026-07-09

**Authors:** Yuwei Shi, Xinmei Li, Yufan Hao, Qiaoyu Wu, Yuji Yu, Siyu Li, Nuo Xu, Yi‐Hsuan Wu, Eunhye Lee, Chi Chang, Emily Hu, Ying Lu, Elizabeth Delzell, Ann W. Hsing, Shankuan Zhu

**Affiliations:** ^1^ Department of Endocrinology of the Second Affiliated Hospital of Zhejiang University School of Medicine Chronic Disease Research Institute School of Public Health School of Medicine Zhejiang University Hangzhou Zhejiang China; ^2^ Department of Nutrition and Food Hygiene School of Public Health School of Medicine Zhejiang University Hangzhou Zhejiang China; ^3^ Stanford Prevention Research Center Department of Medicine Stanford School of Medicine Stanford University Stanford California USA; ^4^ Office of Medical Education Research and Development College of Medicine Michigan State University East Lansing Michigan USA; ^5^ Department of Biomedical Data Science Stanford School of Medicine Stanford University Stanford California USA; ^6^ Department of Epidemiology and Population Health Stanford School of Medicine Stanford University Stanford California USA; ^7^ Stanford Cancer Institute Stanford School of Medicine Stanford University Stanford California USA

**Keywords:** aging, biological age acceleration, gut microbiota, sweetened beverage

## Abstract

Although substantial evidence links sweetened beverage (SB) intake to chronic diseases, its association with biological aging and underlying mechanisms remains unclear. This population‐based study included 9104 adults aged 18–80 years from the WELL‐China cohort. SB consumption was assessed using a validated food frequency questionnaire. Biological age and biological age acceleration (BAacc) were estimated using the Klemera and Doubal method (KDM). After multivariable adjustment, frequent SB consumption was significantly associated with accelerated KDM‐derived BAacc, compared with non‐consumers (β = 0.29 years, 95% CI: 0.06–0.52). This association was stronger among participants younger than 55 years (*P* for interaction < 0.05). Gut microbiota was profiled using 16S rRNA gene sequencing. We identified 10 genera significantly associated with the frequent SB consumption, and 56 genera significantly associated with BAacc. Notably, five genera (*Allisonella*, *Lactobacillus*, *Weissella*, *Lactococcus*, and *Bacilli_unclassified*) were shared between the two analyses and enriched in both frequent SB consumers and individuals with higher BAacc. Frequent SB consumption is associated with accelerated biological aging, especially among adults younger than 55 years. Shared gut microbial signatures associated with both SB intake and BAacc suggest a potential microbiota‐related pathway. These findings support reducing SB consumption as a potential strategy for promoting healthy aging.

## Graphical abstract

Frequent sweetened beverage consumption is associated with accelerated biological aging in a large Chinese population. Five gut microbial genera are consistently enriched in both frequent sweetened beverage consumers and individuals with accelerated aging and show significant mediating effects, suggesting a potential microbiota‐related pathway linking sweetened beverage intake to biological aging.

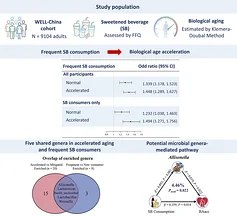

## 1 Introduction

Human biological aging i driven by a complex interplay of genetic, dietary, and environmental factors, making chronological age an imperfect proxy for the true aging process [1, 2]. This has led to growing interest in the concept of biological age (BA), which reflects the functional decline across multiple physiological systems [3, 4]. Among various approaches to BA estimation, the Klemera‐Doubal Method (KDM) identifies a panel of age‐related biomarkers and integrates their values into an algorithm capable of capturing multisystem physiological dysregulation [5, 6, 7]. KDM‐derived BA is cost‐effective and well suited for large‐scale population studies [8].

In recent years, the consumption of sweetened beverages (SB) in China has been increasing, particularly among younger populations [9]. Previous studies have shown that frequent intake of SB, including both sugar‐sweetened and artificially sweetened types, is associated with a range of aging‐related diseases, such as metabolic syndrome, type 2 diabetes, and chronic kidney disease [10, 11, 12, 13]. Mechanistically, sugars in these beverages, especially fructose, may accelerate the biological aging process by promoting inflammation, inducing oxidative stress, and altering the composition of the gut microbiota [14]. However, despite the widespread consumption of SB, evidence directly linking their intake to biological aging remains limited. Moreover, the potential mediating role of the gut microbiota in this relationship has not yet been elucidated.

In this study, we utilized the KDM method to calculate BA and estimate biological aging status based on composite clinical biomarkers and examined the association between SB consumption and accelerated biological aging. Given that emerging evidence suggests that SB intake may be associated with alterations in gut microbiota composition, and that gut microbiota profiles have been linked to aging‐related processes [15, 16, 17], we further explored whether shared microbial signatures might underlie the relationship between SB consumption and biological age acceleration (BAacc).

## 2 Methods

### 2.1 Study Population

The Wellness Living Laboratory China (WELL‐China) was a prospective population‐based cohort study that enrolled a total of 10 268 participants aged 18–80 years between 2016 and 2019 [18]. Participants with missing data on clinical biomarkers, SB intake, or covariates, as well as those who self‐reported cancer or had implausible energy intake (<800 or >4000 kcal/day for males, <500 or >3500 kcal/day for females), were excluded. A total of 9104 participants were included in the final analysis. For gut microbiota analyses, participants without fecal samples or information on antibiotic use were further excluded, resulting in a final subsample of 6375 individuals. The detailed flow of participant selection is presented in Figure 1. Ethical approval was obtained from the Institutional Review Boards of Zhejiang University and Stanford University, and all the participants provided their written informed consent.

**FIGURE 1:**
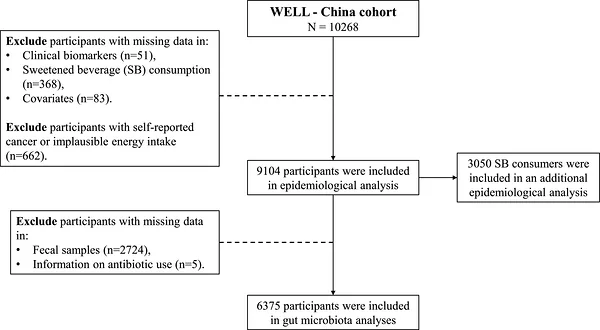
Flowchart of participant selection in the study.

### 2.2 Calculation of Biological Age and Biological Age Acceleration

BA was estimated using the KDM, which integrates multiple clinical and anthropometric biomarkers reflecting diverse physiological systems [7]. Thirteen biomarkers were selected for BA estimation: alkaline phosphatase (ALP), albumin (ALB), blood urea nitrogen (BUN), creatinine (CR), systolic blood pressure (SBP), forced expiratory volume in one second (FEV_1_), fasting blood glucose (FBG), body mass index (BMI), platelet count (PLT), white blood cell count (WBC), glutamic oxaloacetic transaminase (GOT), gamma‐glutamyl transpeptidase (GGT), and lactate dehydrogenase (LDH), which were previously used and validated in a large Chinese cohort [19]. Detailed information on these biomarkers is provided in Table S1, and the correlations between these biomarkers are shown in Figure S1.

The KDM algorithm was implemented using the BioAge package in R software [20]. More details are presented in the Supporting Method. BAacc was defined as the residual of BA regressed on chronological age, representing the deviation of biological age from chronological expectations. A positive BAacc indicates accelerated biological aging, whereas a negative BAacc reflects decelerated aging. Given the physiological differences between sexes, BA and BAacc were estimated separately for men and women [21]. BAacc was analyzed both as a continuous variable and as a categorical variable reflecting three biological aging statuses: mitigated aging (BAacc < −1), normal aging (−1 ≤ BAacc ≤ 1), and accelerated aging (BAacc > 1), using cutoffs consistent with previous studies and approximately corresponding to ±1 standard deviation [21].

### 2.3 Assessment of Sweetened Beverage Consumption

Information on SB consumption was obtained through a validated food frequency questionnaire (FFQ) using the question: “How often have you consumed sweetened beverages (such as cola, Sprite, sports drinks, etc.) in the past year?” [22]. Participants were categorized into four groups based on consumption frequency: non‐consumers, rare consumers (less than once per week), occasional consumers (1–2 times per week), and frequent consumers (3 or more times per week). Additionally, dietary energy and carbohydrate intake were estimated based on the China Food Composition Table (Standard Edition) [23].

### 2.4 Fecal Samples Collection, Microbial DNA Extraction, and 16S rRNA Gene Profiling

Fecal samples were self‐collected by participants following a standardized study protocol, either on the morning of their physical examination day or the evening prior. Upon receiving the samples at the medical examination center, the staff immediately stored them on dry ice for cold storage. Subsequently, the samples were transported to our laboratory within 4 h and stored at −80°C until testing [24].

Following sequencing, raw FASTQ files of the 16S rRNA gene were processed using the Quantiative Insights Into Microbial Ecology 2 platform (QIIME 2, version 2022.2) [25]. Raw sequences were demultiplexed and imported into QIIME 2 using the q2‐demux plugin, followed by denoising with the DADA2 plugin. The DADA2 pipeline was applied to perform quality filtering, trimming of low‐quality regions, and removal of chimeric sequences, resulting in the generation of amplicon sequence variants (ASVs), which were summarized into a feature table. Taxonomic classification of ASVs was performed using a naïve Bayes classifier implemented in the q2‐feature‐classifier plugin (classify‐sklearn), trained on the SILVA 138 database (99% OTUs) [26].

Alpha diversity indices were calculated using the q2‐diversity plugin based on rarefied ASV counts (minimum sequencing depth of 10 000 reads). Genus‐level abundance tables were generated from the feature table and converted to relative abundance by normalizing to the total counts per sample for downstream comparative analyses of gut microbiota composition. Genera with a prevalence below 10% and a relative abundance below 0.0001% were excluded, leaving 188 genera for subsequent analyses. The relative abundances of genera were log‐transformed prior to analysis to improve normality.

### 2.5 Covariates

Demographic and lifestyle information was obtained through face‐to‐face interviews using a standardized questionnaire. Education level was classified as elementary (illiterate or elementary school), secondary (junior high or high/vocational school), and higher (college or above). Smoking and drinking status were categorized as current or non‐current. Tea and coffee consumption were grouped as never, occasional, or frequent. Physical activity was assessed using the short form of the International Physical Activity Questionnaire (IPAQ) and classified into low, moderate, or high levels [27]. Cardiovascular disease (CVD) was defined as self‐reported history of at least one of the following conditions: coronary heart disease, atherosclerosis, arrhythmia, heart failure, stroke, myocardial infarction, or other cardiovascular disease. Kidney disease was also defined based on self‐reported history. Diabetes mellitus was defined using both self‐reported history and objective clinical measurements, including fasting plasma glucose ≥ 7.0 mmol/L or glycosylated hemoglobin (HbA1c) ≥ 6.5% at baseline. Antibiotic use was assessed through face‐to‐face questionnaires, in which participants were asked whether they had used antibiotics within the past three months. Antibiotic use was included as a binary variable (yes/no).

### 2.6 Statistical Analyses

Continuous variables were summarized as means (standard deviations, SD) and categorical variables as counts (percentages). Group differences were assessed using the chi‐square test for categorical variables, one‐way ANOVA for normally distributed continuous variables, and the Kruskal‐Wallis test for non‐normally distributed variables.

The association between SB consumption (main predictor) and BAacc (outcome, continuous variable) was evaluated using multivariable linear regression. The association between SB consumption (main predictor) and biological aging status (outcome; categorized as mitigated, normal, and accelerated aging) was further examined using multinomial logistic regression, with mitigated aging as the reference group. The covariates included age, sex, education level, physical activity, smoking status, drinking status, tea and coffee consumption, dietary energy intake, CVD, kidney disease, and diabetes mellitus. Analyses were conducted in the total population (*n* = 9104) and among SB consumers only (*n* = 3050) to minimize potential bias due to age differences between consumers and non‐consumers.

Interaction and stratified analyses were conducted to examine whether the associations between SB consumption and BAacc differed by age, sex, and education level. Multiplicative interaction terms between SB consumption and subgroup variables were included in the fully adjusted multivariable linear regression models in the total population and among SB consumers only. Age was dichotomized at 55 years, corresponding to the median age of the study population and a commonly used threshold to distinguish midlife from older adulthood [28]. Stratified analyses were subsequently performed in the total population and among SB consumers only.

In addition, sensitivity analyses were conducted by excluding participants with major chronic diseases at baseline and by using the normal aging group as the reference category to assess the robustness of the primary findings.

In gut microbiota analyses (*n* = 6375), associations of SB consumption and biological aging status (main predictor, analyzed separately) with gut microbial α‐diversity indices (outcomes: Shannon index, Pielou's evenness, and Faith's phylogenetic diversity) were assessed using multivariable linear regression, adjusting for the same covariates as above and antibiotic use. Differences in overall microbial composition (β‐diversity) were evaluated using permutational multivariate analysis of variance (PERMANOVA; 999 permutations, Bray‐Curtis distance), with SB consumption or biological aging status as the main predictor and the same covariates included. At the genus level, associations were assessed using *MaAsLin2*, with microbial genera as outcomes and SB consumption and biological aging status as the main predictors (analyzed separately), adjusting for the same covariates. To account for multiple testing, *p* values were corrected using the Benjamini‐Hochberg false discovery rate (FDR) method, with an FDR < 0.1 considered statistically significant [15, 29]. In mediation analyses, SB consumption levels (with non‐consumers as the reference group) were included as the independent variable; each overlapping genus was analyzed separately as a mediator, and continuous BAacc was treated as the outcome. Mediation effects were estimated using 1000 simulations. Genera with both significant average causal mediation effects (ACME) and average direct effects (ADE) (*p* < 0.05) were considered to exhibit statistically significant mediation.

Additional analyses were conducted to examine the associations between SB consumption and individual biomarkers included in the biological age model using multivariable linear regression. Furthermore, associations between the five overlapping genera and these biomarkers were assessed using multivariable linear regression models.

All statistical analyses above were performed with R version 4.2.3. A two‐sided *p* < 0.05 was considered statistically significant.

## 3 Results

### 3.1 Characteristics of Study Participants

A total of 9104 participants were included in the analysis, with a mean (±SD) age of 54.2 ± 13.5 years, and 61.6% were women. Baseline characteristics of participants across biological aging status are shown in Table 1. Among them, 33.7%, 37.6%, and 28.8% were categorized into the mitigated, normal, and accelerated aging groups, respectively. Participants with accelerated aging tended to be men, have lower education levels, and higher prevalence of cardiovascular diseases and diabetes compared with those with mitigated or normal aging. They were also more likely to be frequent consumers of SB, while differences in smoking, alcohol intake, and dietary factors were minimal across groups. Baseline characteristics of participants across SB consumption are shown in Table S2.

**TABLE 1:** Baseline characteristics of study participants according to biological aging status.

	Mitigated Aging (Baacc < ‐1)	Normal (|BAacc ≤ 1)	Accelerated Aging (BAacc > 1)	*p*‐value
**Total participants**	3064	3422	2618	
**Age (years)**	54.75 (13.31)	53.53 (13.57)	54.31 (13.49)	<0.001
**Sex (%)**				<0.001
Male	1191 (38.87)	1220 (35.65)	1084 (41.41)	
Female	1873 (61.13)	2202 (64.35)	1534 (58.59)	
**SB consumption (%)**				0.001
Non‐consumer	2047 (66.81)	2224 (64.99)	1783 (68.11)	
Rare consumer	701 (22.88)	830 (24.25)	536 (20.47)	
Occasional consumer	207 (6.76)	205 (5.99)	164 (6.26)	
Frequent consumer	109 (3.56)	163 (4.76)	135 (5.16)	
**Education level (%)**				<0.001
Elementary	479 (15.63)	692 (20.22)	760 (29.03)	
Secondary	1684 (54.96)	1889 (55.20)	1371 (52.37)	
Higher	901 (29.41)	841 (24.58)	487 (18.60)	
**Physical activity (%)**				0.005
Light	492 (16.06)	590 (17.24)	449 (17.15)	
Moderate	1601 (52.25)	1676 (48.98)	1241 (47.40)	
Heavy	971 (31.69)	1156 (33.78)	928 (35.45)	
**Smoking status (%**)				0.377
Non‐current	2519 (82.21)	2802 (81.88)	2116 (80.83)	
Current	545 (17.79)	620 (18.12)	502 (19.17)	
**Drinking status (%)**				0.343
Non‐current	1672 (54.57)	1929 (56.37)	1457 (55.65)	
Current	1392 (45.43)	1493 (43.63)	1161 (44.35)	
**Tea consumption (%)**				0.124
Never	997 (32.54)	1183 (34.57)	922 (35.22)	
Occasional	801 (26.14)	915 (26.74)	683 (26.09)	
Frequent	1266 (41.32)	1324 (38.69)	1013 (38.69)	
**Coffee consumption (%)**				<0.001
Never	1936 (63.19)	2287 (66.83)	1859 (71.01)	
Occasional	921 (30.06)	932 (27.24)	649 (24.79)	
Frequent	207 (6.76)	203 (5.93)	110 (4.20)	
**Dietary energy intake (kcal)**	1370.70 (520.24)	1355.96 (528.63)	1379.83 (533.64)	0.204
**Cardiovascular disease (%)**	198 (6.46)	195 (5.70)	155 (5.92)	0.421
**Diabetes mellitus (%)**	119 (3.89)	172 (5.04)	280 (10.72)	<0.001
**Kidney disease (%)**	72 (2.35)	88 (2.57)	85 (3.25)	0.099

Continuous variables are presented as means ± standard deviations (SD), and categorical variables are presented as numbers (percentages). Differences across biological aging groups were assessed using the chi‐square test for categorical variables. For continuous variables, one‐way analysis of variance (ANOVA) was applied to normally distributed variables, whereas the Kruskal‐Wallis test was used for non‐normally distributed variables. Biological aging status was defined based on BAacc as follows: mitigated aging (BAacc < −1), normal aging (−1 ≤ BAacc ≤ 1), and accelerated aging (BAacc > 1).

### 3.2 Association of SB Consumption With Biological Aging

Multivariable linear regression analyses examining the association between SB consumption and BAacc are presented in Table 2. In the total population (*n* = 9,104), compared with non‐consumers, frequent SB consumers showed significantly higher BAacc in the fully adjusted model (β = 0.307, 95% CI: 0.072–0.542), whereas no significant associations were observed for rare or occasional consumers. Among SB consumers (*n* = 3050), using rare consumers as the reference group, frequent consumers remained significantly associated with higher BAacc (β = 0.333, 95% CI: 0.097–0.569). Results from multinomial logistic regression analyses showed that, compared with non‐consumers, frequent SB consumers had higher odds of accelerated aging (OR = 1.448, 95% CI: 1.289–1.627), while no significant associations were observed for rare or occasional consumers (Table 3). Among SB consumers, frequent consumers also had higher odds of accelerated aging compared with rare consumers (OR = 1.494, 95% CI: 1.271–1.756). Similar patterns were observed for normal aging status.

**TABLE 2:** Associations between sweetened beverage (SB) consumption and biological age acceleration (BAacc).

	Non‐consumer	Rare (<1 serving/week)	Occasional (1‐2 servings/week)	Frequent (≥3 servings/week)	*P*‐trend
**All participants (*n* = 9104)**	6054	2067	576	407	
Fully adjusted model	Ref.	0.027 (−0.092, 0.146)	0.058 (−0.141, 0.258)	0.307 (0.072, 0.542)*	0.034
**SB consumers (*n* = 3050)**	—	2067	576	407	
Fully adjusted model	—	Ref.	0.061 (−0.138, 0.260)	0.333 (0.097, 0.569)*	0.010

**TABLE 3:** Associations between sweetened beverage (SB) consumption and biological aging status.

	Non‐consumer	Rare (<1 serving/week)	Occasional (1–2 servings/week)	Frequent (≥3 servings/week)	*P*‐trend
**All participants (*n* = 9104)**	6054	2067	576	407	
Normal	Ref.	1.113 (0.990, 1.251)	0.924 (0.817, 1.045)	1.339 (1.178, 1.523)*	0.104
Accelerated	Ref.	1.010 (0.887, 1.151)	1.032 (0.928, 1.148)	1.448 (1.289, 1.627)*	0.047
**SB consumers (*n* = 3050)**	—	2067	576	407	
Normal	—	Ref.	0.827 (0.676, 1.013)	1.232 (1.038, 1.463)*	0.506
Accelerated	—	Ref.	1.049 (0.844, 1.303)	1.494 (1.271, 1.756)*	0.013

Multivariable linear regression models were applied to evaluate the associations between SB consumption and BAacc. Coefficients and 95% confidence intervals (CIs) are presented. Fully adjusted models included age, sex, education level, physical activity, smoking status, drinking status, tea and coffee consumption, dietary energy intake, cardiovascular disease, diabetes mellitus, and kidney disease. In the analysis of all participants, non‐consumers were used as the reference group; in the analysis of SB consumers, rare consumers (<1 serving/week) were used as the reference group. ^*^ *p* < 0.05

Multinomial logistic regression models were applied to evaluate the associations between SB consumption and biological aging status. Odds ratios (ORs) and 95% confidence intervals (CIs) are presented. The mitigated aging group was used as the reference category. Models were adjusted for age, sex, education level, physical activity, smoking status, drinking status, tea and coffee consumption, dietary energy intake, cardiovascular disease, diabetes mellitus, and kidney disease. ^*^ *p* < 0.05Significant interaction effects were observed between SB consumption and age in the total population (*P* for interaction = 0.013), whereas no significant interactions were observed for sex or education level (*P* for interaction > 0.05) (Figure 2). Subgroup analyses showed that the positive association between frequent SB consumption and BAacc was more evident among participants younger than 55 years (β = 0.414, 95% CI: 0.147–0.680), females (β = 0.487, 95% CI: 0.162–0.811), and individuals with higher education levels (β = 0.528, 95% CI: 0.159–0.897) in the total population. Similar patterns were observed among SB consumers only.

**FIGURE 2:**
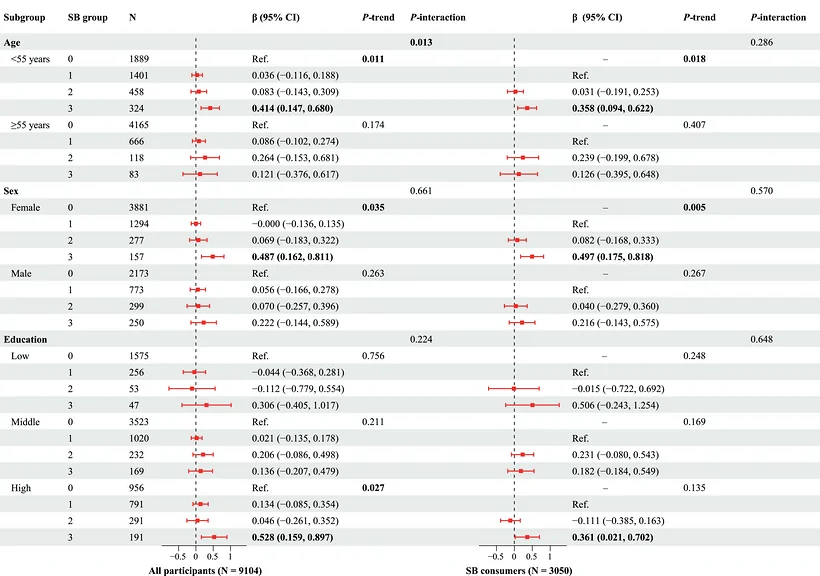
Subgroup analyses of the association between sweetened beverage (SB) consumption and biological age acceleration (BAacc).

Significant interaction effects were observed between SB consumption and age in the total population (*P* for interaction = 0.013), whereas no significant interactions were observed for sex or education level (*P* for interaction > 0.05) (Figure 2). Subgroup analyses showed that the positive association between frequent SB consumption and BAacc was more evident among participants younger than 55 years (β = 0.414, 95% CI: 0.147–0.680), females (β = 0.487, 95% CI: 0.162–0.811), and individuals with higher education levels (β = 0.528, 95% CI: 0.159–0.897) in the total population. Similar patterns were observed among SB consumers only.

Subgroup analyses were conducted using multivariable linear regression models to examine the association between SB consumption and BAacc across different population subgroups. Models were adjusted for age, sex, physical activity, education level, smoking status, drinking status, tea and coffee consumption, dietary energy intake, cardiovascular disease, diabetes mellitus, and kidney disease. Interaction terms between SB consumption and subgroup variables (age, sex, and education) were included in the models to formally test effect modification, and corresponding *P*‐interaction values were reported. SB consumption categories were defined as follows: 0, non‐consumers; 1, <1 serving/week; 2, 1–2 servings/week; 3, ≥3 servings/week.

In sensitivity analyses, after excluding participants with major chronic diseases, the associations remained materially unchanged. Frequent SB consumption was still significantly associated with higher BAacc in multivariable linear regression models (Table S3), both in the overall sample and among SB consumers. Consistently, in multinomial logistic regression models, frequent SB consumption was associated with higher odds of accelerated aging (vs. mitigated aging), both in the overall sample and among SB consumers (Table S4). In addition, sensitivity analyses using the normal aging group as the reference category yielded largely consistent results (Table S5). Among all participants, frequent SB consumption remained associated with lower odds of mitigated aging and higher odds of accelerated aging. Similar patterns were observed among SB consumers, with frequent consumers showing higher odds of accelerated aging.

### 3.3 Gut Microbial Signatures in Relation to SB Consumption and Biological Aging

We first evaluated the association between the gut microbiota and biological aging status. The accelerated aging group exhibited significantly reduced alpha‐diversity compared to the mitigated aging group, as evidenced by a lower Shannon index (*p* = 0.012), Pielou's evenness (*p* = 0.016), and Faith PD (*p* = 0.028; Figure 3A). Consistent with this, BAacc showed an inverse correlation with three alpha‐diversity indices (all *p* < 0.05; Figure S2). Although no significant differences in alpha‐diversity were observed across SB consumption levels (Figure 3B), Beta‐diversity analysis revealed distinct microbial structural shifts. Specifically, significant clustering was observed between the accelerated and mitigated aging groups (*p* < 0.001; Figure 3C), as well as between frequent SB consumers and non‐consumers (*p* < 0.001; Figure 3D).

**FIGURE 3:**
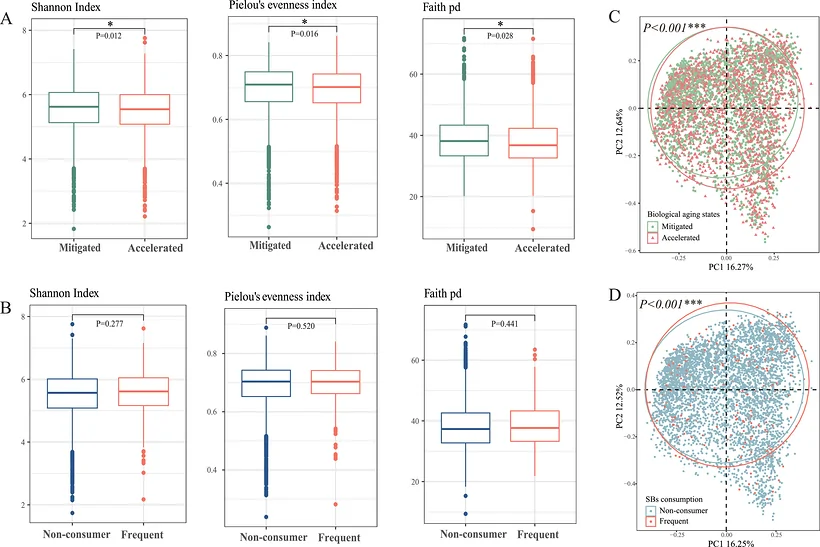
Associations of biological aging status and sweetened beverage (SB) consumption with gut microbial α‐ and β‐diversity (A, B) Multivariable linear regression models were applied to evaluate the associations of biological aging status (A) and SB consumption (B) with gut microbial α‐diversity indices. (C, D) Gut microbial β‐diversity was evaluated using principal coordinate analysis (PCoA) based on the Bray‐Curtis dissimilarity matrix at the genus level. Differences in microbial community structure across biological aging groups (accelerated aging vs. mitigated aging) (C) and SB consumption groups (frequent consumers vs. non‐consumers) (D) were assessed using permutational multivariate analysis of variance (PERMANOVA; 999 permutations). All models were adjusted for age, sex, physical activity, education level, smoking status, drinking status, tea and coffee consumption, dietary energy intake, cardiovascular disease, kidney disease, diabetes mellitus, and antibiotic use. ^*^ *p* < 0.05; ^**^ *p* < 0.01; ^***^ *p* < 0.001.

To further identify specific microbial contributors, MaAsLin2 analyses were performed. We identified 56 genera significantly associated with accelerated aging at FDR < 0.10, of which 39 remained significant at FDR < 0.05 (Figure 4A). We also identified 10 genera associated with frequent SB consumption at FDR < 0.10, of which eight remained significant at FDR < 0.05 (Figure 4B). Notably, five overlapping genera, *Allisonella*, *Lactobacillus*, *Weissella*, *Lactococcus*, and *Bacilli_unclassified*, were consistently enriched in both the accelerated aging and frequent SB consumption groups (Figure 4C). Mediation analysis was subsequently conducted to examine their potential role in the SB‐BAacc association. All five genera showed significant mediation effects, with *Allisonella*, *Lactobacillus*, *Bacilli_unclassified*, *Weissella*, and *Lactococcus* mediating 5.49%, 4.66%, 4.46%, 4.40%, and 4.13% of the total association, respectively (all *p* < 0.05; Figure 4D).

**FIGURE 4:**
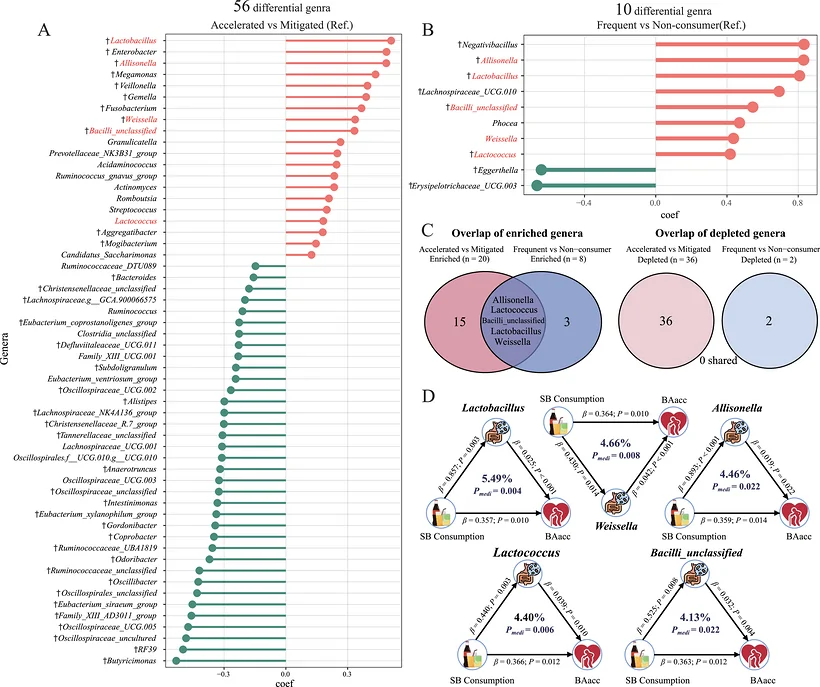
Shared gut microbial signatures associated with accelerated biological aging and frequent sweetened beverage (SB) consumption. (A) 56 genera were significantly associated with biological aging status groups (accelerated aging vs. mitigated aging) at FDR < 0.1. (B) 10 genera were significantly associated with SB consumption (frequent consumers vs. non‐consumers) at FDR < 0.1. Genera marked with † in the upper‐left corner remained significant at FDR < 0.05; unmarked genera met the exploratory threshold of FDR < 0.1 only. (C) Five overlapping genera were identified in both the accelerated aging group and the frequent SB consumption group, representing descriptive overlap between the two analyses; no depleted genera were shared between the comparisons. (D) Mediation analyses of the five overlapping genera in the association between frequent SB consumption and BAacc. All models were adjusted for age, sex, physical activity, education level, smoking status, drinking status, tea and coffee consumption, dietary energy intake, cardiovascular disease, kidney disease, diabetes mellitus, and antibiotic use.

### 3.4 SB Consumption, Identified Genera, and Biological Aging‐Related Biomarkers

Additional analyses showed that SB consumption was significantly associated with several biomarkers included in the biological age model (Table S6). In particular, frequent SB consumption was positively associated with BMI, WBC, and LDH (all *p* < 0.001), as well as ALP and BUN. Figure S3 shows the associations between the five overlapping genera and these biomarkers. *Allisonella* was positively associated with several metabolic and inflammatory markers, including BMI, WBC, SBP, and FBG, whereas *Weissella* was positively associated with BMI and LDH and inversely associated with PLT and ALB (all p < 0.05). *Lactococcus* also showed a positive association with BMI and inverse associations with FEV_1_ and ALB.

## 4 Discussion

In this large population‐based study, we found that frequent consumption of SB was significantly associated with BAacc, independent of chronological age. The association was particularly evident among younger adults. Moreover, we identified five overlapping microbial signatures between frequent SB consumers and individuals with biological age acceleration, with consistent directions of association. Together, these findings provide additional evidence that higher SB intake may be associated with accelerated biological aging, potentially in relation to gut microbial composition.

Evidence linking SB consumption to biological aging remains limited. A prior cross‐sectional analysis of US adults using NHANES data reported a positive association between sugar‐sweetened beverage intake and phenotypic age acceleration [30]. However, substantial differences exist in sweetened beverage consumption patterns and amounts between Chinese and U.S. populations, and the aging process itself may vary across ethnicities. This study is the first to demonstrate a positive association between SB consumption and accelerated biological aging in the Chinese population. Consistent associations have also been reported using alternative aging indicators, such as telomere length, similarly supporting the relevance of SB consumption to aging‐related physiological processes [31].

A significant age‐specific association was observed. Subgroup analyses showed that the positive association between SB consumption and BAacc was significant among younger (< 55 years) participants, whereas no significance was observed in older (≥ 55 years) populations. Consistent with this, previous studies have reported a 43% surge in SB‐related mortality among Chinese populations aged 15–49, alongside higher rates of type 2 diabetes‐related years of life with disability and disability‐adjusted life years in young and middle‐aged males compared to their older counterparts [32, 33]. The SB consumer population in China tends to be younger, and older people drink SB more infrequently due to their traditional dietary habits and higher awareness of health management [9, 34]. These findings suggest that reducing sugar‐sweetened beverage consumption during younger adulthood is a pivotal window of opportunity for slowing biological aging and mitigating the long‐term burden of age‐related diseases.

Biological aging was assessed using the KDM, a clinical biomarker‐based metric reflecting systemic physiological dysregulation [35]. Compared with other aging measures, such as DNA methylation‐based clocks, KDM captures systemic physiological dysregulation and has shown good predictive ability for mortality and health outcomes [36]. However, different aging metrics capture distinct aspects of the aging process, and KDM may be more closely linked to clinical and metabolic alterations rather than molecular aging signatures [37]. Therefore, integrating multiple measures offers a more comprehensive biological perspective.

The pervasive appeal of SB is largely driven by their high sugar content, particularly sucrose and fructose, which enhances palatability and may promote habitual overconsumption through reinforcement of sweet preference [14, 38, 39]. Although most existing studies linking sugars to gut microbiota are based on animal models, emerging human evidence also suggests that SB intake and dietary sugar intake may alter gut microbiota composition [15, 40, 41]. One recent population‐based study identified nine gut microbial species significantly associated with SB intake and further showed that these associations were largely driven by fructose and glucose intake from SB [15]. Another human study reported that high‐fructose corn syrup intake was associated with reduced abundance of *Erysipelatoclostridium* [40]. Consistently, we identified 10 genera significantly associated with SB consumption, including a reduced abundance of *Erysipelotrichaceae_UCG‐003*, a closely related genus within the same bacterial family. In addition, recent experimental evidence suggests that high‐dose fructose intake may overwhelm intestinal fructose absorption and clearance capacity, allowing fructose to reach both the liver and the colonic microbiota, which may partly explain why SB consumption could be associated with gut microbial alterations [42].

Notably, we identified five overlapping genera that were associated with both frequent SB consumption and biological age acceleration. These genera also showed significant mediation effects, suggesting that they may serve as candidate microbial features related to the link between SB consumption and biological aging. Among them, *Allisonella*, *Lactobacillus*, *Weissella*, and *Lactococcus* belong to the Firmicutes phylum, which has previously been associated with high‐fructose diets and metabolic disturbances, such as obesity [43, 44, 45]. Specifically, *Allisonella* has been associated with increased frailty risk, and emerging evidence suggests a potential association with sarcopenia and obesity, indicating its relevance to aging‐related decline [46, 47]. In our study, *Allisonella* also showed strong positive associations with BMI, FPG, and SBP. In addition, *Weissella* is a lactic acid bacterium with both probiotic potential and opportunistic pathogenic characteristics. In our data, *Weissella* was positively associated with BMI and LDH and inversely associated with PLT [48]. We also observed that SB consumption was significantly associated with several of these biomarkers, particularly BMI and LDH. Excessive SB intake may contribute to positive energy balance and metabolic dysfunction, including obesity and metabolic syndrome, which have been linked to accelerated biological aging [49, 50]. Taken together, these findings suggest that SB consumption may be linked to biological aging partly through microbiota‐related metabolic pathways involving biomarkers such as BMI, although further longitudinal and mechanistic studies are needed to clarify these associations.

Human gut‐derived bacterial genomes harbor abundant prophages [51]. Recent studies have further isolated and characterized inducible temperate phages from human gut bacteria, supporting their role in shaping microbial community structure [52]. Notably, sweeteners and other dietary compounds may influence the gut microbiome not only through direct effects on bacterial growth but also by modulating prophage induction and phage particle production in lysogenic bacteria, thereby indirectly altering bacterial communities [53]. This evidence hints at a possible phage‐mediated mechanism for how SB consumption may alter gut microbes, though our sequencing data cannot assess gut virome or phage activity.

There are several strengths in our study. To the best of our knowledge, this is the first population‐based study to explore the associations between SB consumption, gut microbiota, and BAacc. By employing a validated and modifiable aging metric, our study provides additional insights into potential nutrition‐oriented strategies for healthy aging. The observed gut microbial signatures may reflect microbiota‐related pathways associated with biological aging.

However, several limitations exist. First, our study was a cross‐sectional study and thus could not prove causality. Second, our FFQ did not distinguish sugar‐sweetened from non‐sugar‐sweetened beverages, limiting analyses by beverage type. This distinction is important because non‐sugar sweeteners may affect gut microbiota composition, although human evidence remains inconsistent [54, 55, 56]. However, in China, sugar‐sweetened beverages are still more commonly consumed than non‐sugar‐sweetened beverages, but the latter are increasingly popular [57]. Third, the information on SB consumption was self‐reported, potentially introducing the risk of recall bias and misclassification. Fourthly, despite our adjustment for a wide range of potential covariates, the inherent nature of observational studies means that residual confounding from unobserved factors cannot be entirely ruled out. Fifthly, our analyses were based on a complete‐case approach, which may introduce selection bias. However, the proportion of missing data was low, particularly for covariates. Sixthly, gut microbiota was profiled using 16S rRNA gene sequencing, which limits species‐level resolution and functional annotation.

## 5 Conclusions

In summary, high‐frequency consumption of SB was positively associated with BAacc, particularly among individuals under 55 years. Gut microbiota signatures were also associated with both SB consumption and BAacc, suggesting that these signatures may play a potential role in these associations. These findings highlight the importance of public health strategies aimed at reducing sweetened beverage consumption

## Author Contributions

**Yuwei Shi** and **Shankuan Zhu** designed the study and were responsible for the manuscript. Yuwei Shi and **Xinmei Li** analyzed data. Yuwei Shi, Xinmei Li, **Yufan Hao**, **Qiaoyu Wu**, **Ann W. Hsing**, and Shankuan Zhu wrote and revised the manuscript. Yuwei Shi, Xinmei Li, Qiaoyu Wu, and **Yuji Yu** created the tables and figures. Shankuan Zhu supervised the whole study. All authors contributed to the critical revisions of the manuscript for intellectual content or data collection. All authors read and revised the manuscript and approved the final version.

## Funding

This work was funded by the Nutrilite Health Institute Wellness Fund; the China Medical Board (CMB) [15‐216]; the Cyrus Tang Foundation [419600‐11102]; and the Hsun K. Chou Fund of Zhejiang University Education Foundation [419600‐11107].

## Conflicts of Interest

The authors declare no conflicts of interest.

## Supporting information




**Supporting file**: advs76460‐sup‐0001‐SuppMat.docx

## Data Availability

The data that support the findings of this study are available from the corresponding author upon reasonable request.

## References

[1] B. K. Kennedy , S. L. Berger , A. Brunet , et al., “Geroscience: Linking Aging To Chronic Disease,” Cell 159, no. 4 (2014): 709–713, 10.1016/j.cell.2014.10.039. PMC4852871 25417146

[2] S. Esposito , A. Gialluisi , A. Di Castelnuovo , et al., “Ultra‐Processed Food Consumption Is Associated With The Acceleration Of Biological Aging In The Moli‐Sani Study,” American Journal of Clinical Nutrition 120, no. 6 (2024): 1432–1440, 10.1016/j.ajcnut.2024.10.006. 39500680

[3] M. R. Hamczyk , R. M. Nevado , A. Barettino , V. Fuster , and V. Andrés , “Biological Versus Chronological Aging,” Journal of the American College of Cardiology 75, no. 8 (2020): 919–930, 10.1016/j.jacc.2019.11.062. 32130928

[4] P. Mamoshina , K. Kochetov , E. Putin , et al., “Population Specific Biomarkers of Human Aging: A Big Data Study Using South Korean, Canadian, and Eastern European Patient Populations,” The Journals of Gerontology: Series A 73, no. 11 (2018): 1482–1490, 10.1093/gerona/gly005. PMC6175034 29340580

[5] Z. Liu , “Development and Validation of 2 Composite Aging Measures Using Routine Clinical Biomarkers in the Chinese Population: Analyses From 2 Prospective Cohort Studies,” Journals of Gerontology Series A, Biological Sciences and Medical Sciences 76, no. 9 (2021): 1627–1632, 10.1093/gerona/glaa238. PMC8521780 32946548

[6] L. Me , “Modeling The Rate Of Senescence: Can Estimated Biological Age Predict Mortality More Accurately Than Chronological Age?,” Journals of Gerontology Series A, Biological Sciences and Medical Sciences 68, no. 6 (2013): 667–674, 10.1093/gerona/gls233. PMC3660119 23213031

[7] P. Klemera and S. Doubal , “A New Approach To The Concept And Computation Of Biological Age,” Mechanisms of Ageing and Development 127, no. 3 (2006): 240–248, 10.1016/j.mad.2005.10.004. 16318865

[8] L. Chen , Y. Zhang , C. Yu , et al., “Modeling Biological Age Using Blood Biomarkers And Physical Measurements In Chinese Adults,” EBioMedicine 89 (2023): 104458, 10.1016/j.ebiom.2023.104458. PMC9941058 36758480

[9] D. Li , D. Yu , and L. Zhao , “Trend Of Sugar‐Sweetened Beverage Consumption And Intake Of Added Sugar In China Nine Provinces Among Adults,” Wei Sheng Yan Jiu= Journal Of Hygiene Research 43, no. 1 (2014): 70–72. 24564114

[10] H. Chen , J. Chen , Y. Cao , et al., “Sugary Beverages And Genetic Risk In Relation To Brain Structure And Incident Dementia: A Prospective Cohort Study,” American Journal of Clinical Nutrition 117, no. 4 (2023): 672–680, 10.1016/j.ajcnut.2023.01.015. 36781080

[11] G. Y. Heo , H. B. Koh , J. T. Park , et al., “Sweetened Beverage Intake and Incident Chronic Kidney Disease in the UK Biobank Study,” JAMA Network Open 7, no. 2 (2024): 2356885, 10.1001/jamanetworkopen.2023.56885. PMC10902724 38416492

[12] S. Shin , S. A. Kim , J. Ha , and K. Lim , “Sugar‐Sweetened Beverage Consumption in Relation to Obesity and Metabolic Syndrome Among Korean Adults: A Cross‐Sectional Study From the 2012–2016 Korean National Health and Nutrition Examination Survey (KNHANES),” Nutrients 10, no. 10 (2018): 1467, 10.3390/nu10101467. PMC6213560 30304842

[13] J. P. Drouin‐Chartier , Y. Zheng , Y. Li , et al., “Changes in Consumption of Sugary Beverages and Artificially Sweetened Beverages and Subsequent Risk of Type 2 Diabetes: Results From Three Large Prospective U.S. Cohorts of Women and Men,” Diabetes Care 42, no. 12 (2019): 2181–2189, 10.2337/dc19-0734. PMC6868459 31582428

[14] L. A. Ertuglu , B. Afsar , A. B. Yildiz , et al., “Substitution of Sugar‐Sweetened Beverages for Other Beverages: Can It Be the Next Step Towards Healthy Aging?,” Current Nutrition Reports 10, no. 4 (2021): 399–412, 10.1007/s13668-021-00372-2. 34595722

[15] Y. Zhang , K. Luo , B. A. Peters , et al., “Sugar‐Sweetened Beverage Intake, Gut Microbiota, Circulating Metabolites, And Diabetes Risk In Hispanic Community Health Study/Study Of Latinos,” Cell Metabolism 37, no. 3 (2025): 578–591, 10.1016/j.cmet.2024.12.004. PMC11885037 39892390

[16] K. Tiihonen , A. C. Ouwehand , and N. Rautonen , “Human Intestinal Microbiota And Healthy Ageing,” Ageing Research Reviews 9, no. 2 (2010): 107–116, 10.1016/j.arr.2009.10.004. 19874918

[17] V. J. Maffei , S. Kim , E. Blanchard , et al., “Biological Aging and the Human Gut Microbiota,” The Journals of Gerontology: Series A 72, no. 11 (2017): 1474–1482, 10.1093/gerona/glx042. PMC5861892 28444190

[18] Y. Min , X. Zhao , R. S. Stafford , et al., “Cohort Profile: Well Living Laboratory In China (Well‐China),” International Journal of Epidemiology 50, no. 5 (2021): 1432–1443, 10.1093/ije/dyaa283. 33712826

[19] S. Wang , C. P. Wen , W. Li , et al., “Development of a Novel Multidimensional Measure of Aging to Predict Mortality and Morbidity in the Prospective MJ Cohort,” The Journals of Gerontology: Series A 78, no. 4 (2023): 690–697, 10.1093/gerona/glac161. 35921680

[20] D. Kwon and D. W. Belsky , “A Toolkit For Quantification Of Biological Age From Blood Chemistry And Organ Function Test Data: BioAge,” GeroScience 43, no. 6 (2021): 2795–2808, 10.1007/s11357-021-00480-5. PMC8602613 34725754

[21] Y. Xiang , H. Xu , H. Chen , et al., “Tea Consumption And Attenuation Of Biological Aging: A Longitudinal Analysis From Two Cohort Studies,” The Lancet Regional Health–Western Pacific 42 (2024): 100955, 10.1016/j.lanwpc.2023.100955.

[22] Y. Ru , N. Wang , Y. Min , et al., “Characterization Of Dietary Patterns And Assessment Of Their Relationships With Metabolomic Profiles: A Community‐Based Study,” Clinical Nutrition 40, no. 5 (2021): 3531–3541, 10.1016/j.clnu.2020.12.006. 33349486

[23] M. Zhang , C. Jin , Y. Ding , et al., “Higher Intake of Fat, Vitamin E‐(β+γ), Magnesium, Sodium, and Copper Increases the Susceptibility to Prostatitis‐Like Symptoms: Evidence From a Chinese Adult Cohort,” Nutrients 14, no. 18 (2022): 3675, 10.3390/nu14183675. PMC9501331 36145052

[24] T. Yatsunenko , F. E. Rey , M. J. Manary , et al., “Human Gut Microbiome Viewed Across Age And Geography,” Nature 486, no. 7402 (2012): 222–227, 10.1038/nature11053. PMC3376388 22699611

[25] E. Bolyen , J. R. Rideout , M. R. Dillon , et al., “Reproducible, Interactive, Scalable And Extensible Microbiome Data Science Using QIIME 2,” Nature Biotechnology 37, no. 8 (2019): 852–857, 10.1038/s41587-019-0209-9. PMC7015180 31341288

[26] N. A. Bokulich , B. D. Kaehler , J. R. Rideout , et al., “Optimizing Taxonomic Classification Of Marker‐Gene Amplicon Sequences With QIIME 2's q2‐Feature‐Classifier Plugin,” Microbiome 6, no. 1 (2018): 90, 10.1186/s40168-018-0470-z. PMC5956843 29773078

[27] C. L. Craig , A. L. Marshall , M. Sjöström , et al., “International Physical Activity Questionnaire: 12‐Country Reliability And Validity,” Medicine & Science in Sports & Exercise 35, no. 8 (2003): 1381–1395, 10.1249/01.MSS.0000078924.61453.FB. 12900694

[28] S. B. Dugani , M. V. Moorthy , C. Li , et al., “Association of Lipid, Inflammatory, and Metabolic Biomarkers With Age at Onset for Incident Coronary Heart Disease in Women,” JAMA Cardiology 6, no. 4 (2021): 437–447, 10.1001/jamacardio.2020.7073. PMC7818181 33471027

[29] Y. Wang , G. C. Chen , Z. Wang , et al., “Dietary Acculturation Is Associated With Altered Gut Microbiome, Circulating Metabolites, and Cardiovascular Disease Risk in US Hispanics and Latinos: Results From HCHS/SOL,” Circulation 150, no. 3 (2024): 215–229, 10.1161/CIRCULATIONAHA.124.069824. PMC11460527 39008559

[30] T. Xia , Q. Yuan , Y. Zhang , and G. Shan , “The Associations Between The Energy And Timing Of Sugar‐Sweetened Beverage Intake And Phenotypic Age Acceleration In Us Adults: A Cross‐Sectional Survey Of NHANES 2007–2010,” BMC Public Health 25, no. 1 (2025): 88, 10.1186/s12889-024-21249-3. PMC11707922 39780125

[31] I. Sohn , C. Shin , and I. Baik , “Associations Of Green Tea, Coffee, And Soft Drink Consumption With Longitudinal Changes In Leukocyte Telomere Length,” Scientific Reports 13, no. 1 (2023): 492, 10.1038/s41598-022-26186-y. PMC9832020 36627320

[32] Y. Jiang , T. Xu , W. Dong , C. Chu , and M. Zhou , “Study On The Death And Disease Burden Caused By High Sugar‐Sweetened Beverages Intake In China From 1990 to 2019,” European Journal of Public Health 32, no. 5 (2022): 773–778, 10.1093/eurpub/ckac067. PMC9527974 36190153

[33] M. Li , X. Li , Y. Zhao , et al., “The Burden Of Ischemic Heart Disease And Type 2 Diabetes Mellitus Attributable To Diet High In Sugar‐Sweetened Beverages In China: An Analysis For The Global Burden Of Disease Study 2017,” Journal of Diabetes 13, no. 6 (2021): 482–493, 10.1111/1753-0407.13132. 33151626

[34] M. A. Zhimin , H. O. Xiaoyan , W. Dongyang , W. Xiuli , S. U. N. Yiliang , and L. I. Haiyan , “Research Progress on the Characteristics, Distribution, and Health Relevance of Chinese Dietary Patterns,” Science and Technology of Food Industry 44, no. 10 (2023): 396–405, 10.13386/j.issn1002-0306.2022060202.

[35] K. Wei , S. Peng , N. Liu , et al., “All‐Subset Analysis Improves the Predictive Accuracy of Biological Age for All‐Cause Mortality in Chinese and U.S. Populations,” The Journals of Gerontology: Series A 77, no. 11 (2022): 2288–2297, 10.1093/gerona/glac081. PMC9923798 35417546

[36] W. J. Hastings , I. Shalev , and D. W. Belsky , “Comparability Of Biological Aging Measures In The National Health And Nutrition Examination Study, 1999–2002,” Psychoneuroendocrinology 106 (2019): 171–178, 10.1016/j.psyneuen.2019.03.012. PMC6599717 30999227

[37] B. Warner , E. Ratner , A. Datta , and A. Lendasse , “A Systematic Review Of Phenotypic And Epigenetic Clocks Used For Aging And Mortality Quantification In Humans,” Aging 16, no. 17 (2024): 12414–12427, 10.18632/aging.206098. PMC11424583 39215995

[38] K. S. Burger , “Frontostriatal And Behavioral Adaptations To Daily Sugar‐Sweetened Beverage Intake: A Randomized Controlled Trial,” The American Journal of Clinical Nutrition 105, no. 3 (2017): 555–563, 10.3945/ajcn.116.140145. PMC5320411 28179221

[39] M. Lutter and E. J. Nestler , “Homeostatic And Hedonic Signals Interact In The Regulation Of Food Intake,” The Journal of Nutrition 139, no. 3 (2009): 629–632, 10.3945/jn.108.097618. PMC2714382 19176746

[40] J. Beisner , A. Gonzalez‐Granda , M. Basrai , A. Damms‐Machado , and S. C. Bischoff , “Fructose‐Induced Intestinal Microbiota Shift Following Two Types of Short‐Term High‐Fructose Dietary Phases,” Nutrients 12, no. 11 (2020): 3444, 10.3390/nu12113444. PMC7697676 33182700

[41] S. Ramne , L. Brunkwall , U. Ericson , et al., “Gut Microbiota Composition In Relation To Intake Of Added Sugar, Sugar‐Sweetened Beverages And Artificially Sweetened Beverages In The Malmö Offspring Study,” European Journal of Nutrition 60, no. 4 (2021): 2087–2097, 10.1007/s00394-020-02392-0. PMC8137620 33030577

[42] C. Jang , S. Hui , W. Lu , et al., “The Small Intestine Converts Dietary Fructose Into Glucose and Organic Acids,” Cell Metabolism 27, no. 2 (2018): 351–361, 10.1016/j.cmet.2017.12.016. PMC6032988 29414685

[43] K. Wang , Y. Zhao , L. Xu , X. Liao , and Z. Xu , “Health Outcomes Of 100% Orange Juice And Orange Flavored Beverage: A Comparative Analysis Of Gut Microbiota And Metabolomics In Rats,” Current Research in Food Science 6 (2023): 100454, 10.1016/j.crfs.2023.100454. PMC9932342 36815996

[44] M. Moughaizel , E. Dagher , A. Jablaoui , et al., “Long‐Term High‐Fructose High‐Fat Diet Feeding Elicits Insulin Resistance, Exacerbates Dyslipidemia And Induces Gut Microbiota Dysbiosis In Whhl Rabbits,” PLoS ONE 17, no. 2 (2022): 0264215, 10.1371/journal.pone.0264215. PMC8865649 35196347

[45] X. M. Li , Q. Lv , Y. J. Chen , L. B. Yan , and X. Xiong , “Association Between Childhood Obesity And Gut Microbiota: 16S rRNA Gene Sequencing‐Based Cohort Study,” World Journal of Gastroenterology 30, no. 16 (2024): 2249, 10.3748/wjg.v30.i16.2249. PMC11056921 38690025

[46] L. Wang , X. He , Z. Zhang , and N. Chen , “Distinct Gut Microbiota Signatures In Older People With Sarcopenic Obesity And Sarcopenia Without Obesity,” Clinical Nutrition 49 (2025): 77–89, 10.1016/j.clnu.2025.04.004. 40252601

[47] Z. Yan , G. Guan , H. Jia , H. Li , S. Zhuoga , and S. Zheng , “The Association Between Gut Microbiota And Accelerated Aging And Frailty: A Mendelian Randomization Study,” Aging Clinical and Experimental Research 37, no. 1 (2025): 82, 10.1007/s40520-025-02971-3. PMC11903541 40074999

[48] W. Ma , X. Liu , Y. Jing , et al., “Weissella: From Beneficial Probiotics to Opportunistic Pathogens—A Review,” Nutrients 17, no. 19 (2025): 3162, 10.3390/nu17193162. PMC12525997 41097239

[49] H. Sa , H. De , M. Nm , and H. Ma , “Fructose Metabolism And Metabolic Disease,” Journal of Clinical Investigation 128, no. 2 (2018): 545–555, 10.1172/JCI96702. PMC5785258 29388924

[50] A. Narasimhan , R. R. Flores , C. D. Camell , D. A. Bernlohr , P. D. Robbins , and L. J. Niedernhofer , “Cellular Senescence In Obesity And Associated Complications: A New Therapeutic Target,” Current Diabetes Reports 22, no. 11 (2022): 537–548, 10.1007/s11892-022-01493-w. PMC10123542 36239841

[51] Z. Pei , Y. Liu , Y. Chen , et al., “A Universe Of Human Gut‐Derived Bacterial Prophages: Unveiling The Hidden Viral Players In Intestinal Microecology,” Gut Microbes 16, no. 1 (2024): 2309684, 10.1080/19490976.2024.2309684. PMC10841027 39679618

[52] S. Dahlman , L. Avellaneda‐Franco , E. L. Rutten , et al., “Isolation, Engineering And Ecology Of Temperate Phages From The Human Gut,” Nature 647, no. 8090 (2025): 698–705, 10.1038/s41586-025-09614-7. PMC12629997 41094135

[53] L. Boling , D. A. Cuevas , J. A. Grasis , et al., “Dietary Prophage Inducers And Antimicrobials: Toward Landscaping The Human Gut Microbiome,” Gut Microbes 11, no. 4 (2020): 721–734, 10.1080/19490976.2019.1701353. PMC7524278 31931655

[54] Y. Sun and B. Xu , “A Critical Review On Effects Of Artificial Sweeteners On Gut Microbiota And Gastrointestinal Health,” Journal of the Science of Food and Agriculture 105, no. 5 (2025): 2737–2747, 10.1002/jsfa.14148. 39878083

[55] A. Conz , M. Salmona , and L. Diomede , “Effect of Non‐Nutritive Sweeteners on the Gut Microbiota,” Nutrients 15, no. 8 (2023): 1869, 10.3390/nu15081869. PMC10144565 37111090

[56] F. J. Ruiz‐Ojeda , J. Plaza‐Díaz , M. J. Sáez‐Lara , and A. Gil , “Effects of Sweeteners on the Gut Microbiota: A Review of Experimental Studies and Clinical Trials,” Advances in Nutrition 10, no. 1 (2019): S31–S48, 10.1093/advances/nmy037. PMC6363527 30721958

[57] Z. Wang , L. Shen , J. Ning , et al., “The Consumption of Non‐Sugar Sweetened and Ready‐to‐Drink Beverages as Emerging Types of Beverages in Shanghai,” Nutrients 16, no. 20 (2024): 3547, 10.3390/nu16203547. PMC11510668 39458541

