# Supplementary material for: Frequent Sweetened Beverage Consumption Is Associated With Accelerated Biological Aging: Evidence From a Population‐Based Study and Gut Microbiota Analysis
Source: Adv Sci (Weinh). 2026 Jul 9;13(54):e76460. doi: 10.1002/advs.76460 (PMC13348652; doi:10.1002/advs.76460)
Supplement: Supplementary file 1 [file ADVS-13-e76460-s001.docx]

**Supplementary files**

**This supplementary file contains the following:**

**Supplementary Method.** Calculation of biological age and biological age acceleration

**Supplementary Table 1.** Biomarkers included in the biological age model

**Supplementary Table 2.** Baseline characteristics of study participants according to sweetened beverage (SB) consumption

**Supplementary Table 3.** Sensitivity analysis of the associations between sweetened beverage (SB) consumption and biological age acceleration (BAacc) after excluding participants with major chronic diseases

**Supplementary Table 4.** Sensitivity analysis of the associations between sweetened beverage (SB) consumption and biological aging status after excluding participants with major chronic diseases

**Supplementary Table 5.** Sensitivity analysis of the associations between sweetened beverage (SB) consumption and biological aging status, using the normal aging group as the reference category

**Supplementary Table 6.** Associations between sweetened beverage (SB) consumption and biomarkers included in the biological age model

**Supplementary Figure 1.** Correlations among biomarkers included in the biological age model

**Supplementary Figure 2.** Associations between gut microbial α-diversity indices and biological age acceleration (BAacc)

**Supplementary Figure 3.** Associations between five overlapping gut microbial genera and biomarkers included in the biological age model

**Supplementary Method.** Calculation of biological age and biological age acceleration

The information of biomarkers was showed in **Supplementary Table 1**, and the equation as follows:

$$BA=\frac{\sum_{j=1}^{m} (x_{j}-q_{j})\left( \frac{k_{j}}{s_{j}^{2}} \right)+\frac{\mathrm{CA}}{s_{\mathrm{BA}}^{2}}}{\sum_{j=1}^{m} \left( \frac{k_{j}}{s_{j}^{2}} \right)+\frac{1}{s_{\mathrm{BA}}^{2}}}$$

Where m represents the number of biomarkers; x represents the value of the biomarker j; the parameters q, k, and s correspond to the regression of biomarker j on CA (k is the regression intercept, q is the regression slope, s is the regression root mean squared error); $s_{\mathrm{BA}}$ represents a scaling factor equal to the square root of the variance in CA explained by the biomarker set. In addition, we calculated biological age acceleration (BAacc) as following:

BAacc=BA-CA

The calculations above can be performed using the “*BioAge*” R package.

**Supplementary Table 1.** Biomarkers included in the biological age model

| **Variables** | **System** | **Mean(±SD)** | **Units** |
| --- | --- | --- | --- |
| Age |  | 54.17 (13.47) | Years |
| Alkaline Phosphatase (ALP) | Liver | 74.55 (21.56) | U/L |
| Albumin (ALB) | Liver | 47.77 (2.63) | g/L |
| Blood Urea nitrogen (BUN) | Kidney | 5.13 (1.32) | mmol/L |
| Creatinine (CR) | Kidney | 66.78 (18.60) | μmol/L |
| Systolic Blood Pressure (SBP) | Cardiovascular | 122.55 (16.94) | mmHg |
| Forced Expiratory Volume in One Second (FEV1) | Lung | 2.39 (0.69) | L |
| Fasting Blood Glucose (FBG) | Metabolic | 5.35 (1.25) | mmol/L |
| Body Mass Index (BMI) | Metabolic | 23.70 (3.25) | kg/m2 |
| Platelet Count (PLT) | Immune | 222.20 (59.83) | 10^9 cells/L |
| White Blood Cell Count (WBC) | Immune | 6.10 (1.54) | 10^9 cells/L |
| Glutamic Oxaloacetic Transaminase (GOT) | Liver | 21.28 (11.08) | U/L |
| Gamma-Glutamyl Transpeptidase (GGT) | Liver | 28.29 (32.37) | U/L |
| Lactate Dehydrogenase (LDH) | Liver | 188.35 (35.05) | U/L |

**Supplementary Table 2.** Baseline characteristics of the study participants according to sweetened beverage (SB) consumption

|  | **Non-consumer** | **Rare**  (<1 serving/week) | **Occasional**  (1-2 servings/week) | **Frequent**  (≥3 servings/week) | ***P*-value** |
| --- | --- | --- | --- | --- | --- |
| **Total participants** | 6054 | 2067 | 576 | 407 |  |
| **Chronological age (years)** | 58.54 (10.96) | 47.43 (13.48) | 42.304 (13.30) | 40.07 (13.71) | <0.001 |
| **Biological age(years)** | 58.46 (11.09) | 47.27 (13.59) | 42.188 (13.54) | 40.31 (13.82) | <0.001 |
| **Biological age acceleration(years)** | -0.09 (2.31) | -0.16 (2.13) | -0.12 (2.22) | 0.24 (2.28) | 0.015 |
| **Sex (%)** |  |  |  |  | <0.001 |
| Male | 2173 (35.89) | 773 (37.40) | 299 (51.91) | 250 (61.43) |  |
| Female | 3881 (64.11) | 1294 (62.60) | 277 (48.09) | 157 (38.57) |  |
| **Education level (%)** |  |  |  |  | <0.001 |
| Elementary | 1575 (26.02) | 256 (12.39) | 53 (9.20) | 47 (11.55) |  |
| Secondary | 3523 (58.19) | 1020 (49.35) | 232 (40.28) | 169 (41.52) |  |
| Higher | 956 (15.79) | 791 (38.27) | 291 (50.52) | 191 (46.93) |  |
| **Physical activity (%)** |  |  |  |  | <0.001 |
| Light | 910 (15.03) | 382 (18.48) | 138 (23.96) | 101 (24.82) |  |
| Moderate | 3083 (50.93) | 1024 (49.54) | 245 (42.53) | 166 (40.79) |  |
| Heavy | 2061 (34.04) | 661 (31.98) | 193 (33.51) | 140 (34.40) |  |
| **Smoking status (%**) |  |  |  |  | <0.001 |
| Non-current | 5058 (83.55) | 1704 (82.44) | 417 (72.40) | 258 (63.39) |  |
| Current | 996 (16.45) | 363 (17.56) | 159 (27.60) | 149 (36.61) |  |
| **Drinking status (%)** |  |  |  |  | <0.001 |
| Non-current | 3581 (59.15) | 1008 (48.77) | 277 (48.09) | 192 (47.17) |  |
| Current | 2473 (40.85) | 1059 (51.23) | 299 (51.91) | 215 (52.83) |  |
| **Tea consumption (%)** |  |  |  |  | <0.001 |
| Never | 2287 (37.78) | 559 (27.04) | 139 (24.13) | 117 (28.75) |  |
| Occasional | 1349 (22.28) | 701 (33.91) | 200 (34.72) | 149 (36.61) |  |
| Frequent | 2418 (39.94) | 807 (39.04) | 237 (41.15) | 141 (34.64) |  |
| **Coffee consumption (%)** |  |  |  |  | <0.001 |
| Never | 4602 (76.02) | 1053 (50.94) | 254 (44.10) | 173 (42.51) |  |
| Occasional | 1197 (19.77) | 850 (41.12) | 268 (46.53) | 187 (45.95) |  |
| Frequent | 255 (4.21) | 164 (7.93) | 54 (9.38) | 47 (11.55) |  |
| **Dietary energy intake (kcal)** | 1357.58 (514.20) | 1321.73 (502.22) | 1448.64 (575.05) | 1639.080 (667.14) | <0.001 |
| **Cardiovascular disease (%)** | 454 (7.50) | 79 (3.82) | 10 (1.74) | 5 (1.23) | <0.001 |
| **Diabetes mellitus (%)** | 524 (8.67) | 39 (1.89) | 4 (0.69) | 4 (0.99) | <0.001 |
| **Kidney disease (%)** | 189 (3.12) | 42 (2.03) | 9 (1.56) | 5 (1.23) | 0.003 |

**Supplementary Table 3.** Sensitivity analysis of the associations between sweetened beverage (SB) consumption and biological age acceleration (BAacc) after excluding participants with major chronic diseases

|  | Non-consumer | Rare  (<1 serving/week) | Occasional  (1-2 servings/week) | Frequent  (≥3 servings/week) | *P*-trend |
| --- | --- | --- | --- | --- | --- |
| **All participants (n = 7496)** | 4723 | 1860 | 537 | 376 |  |
| Fully adjusted model | Ref. | -0.008 (-0.131, 0.115) | 0.024 (-0.178, 0.225) | 0.273 (0.034, 0.512)* | 0.109 |
| **SB consumers (n = 2773)** | - | 1860 | 537 | 376 |  |
| Fully adjusted model | - | Ref. | 0.031 (-0.168, 0.231) | 0.291 (0.053, 0.530)* | 0.031 |

Multivariable linear regression models were applied to evaluate the associations between SB consumption and BAacc. Coefficients and 95% confidence intervals (CIs) are presented. Fully adjusted models included age, sex, education level, physical activity, smoking status, drinking status, tea and coffee consumption, dietary energy intake, cardiovascular disease, diabetes mellitus and kidney disease. In the analysis of all participants, non-consumers were used as the reference group; in the analysis of SB consumers, rare consumers (<1 serving/week) were used as the reference group. **P* < 0.05.

**Supplementary Table 4.** Sensitivity analysis of the associations between sweetened beverage (SB) consumption and biological aging status after excluding participants with major chronic diseases

|  | Non-consumer | Rare  (<1 serving/week) | Occasional  (1-2 servings/week) | Frequent  (≥3 servings/week) | *P*-trend |
| --- | --- | --- | --- | --- | --- |
| **All participants (n = 7496)** | 4723 | 1860 | 537 | 376 |  |
| Normal | Ref. | 1.162 (1.027, 1.314) * | 0.965 (0.843, 1.105) | 1.480 (1.291, 1.696)* | 0.015 |
| Accelerated | Ref. | 1.075 (0.937, 1.235) | 1.109 (0.983, 1.251) | 1.646 (1.459, 1.856)* | 0.002 |
| **SB consumers (n = 2773)** | - | 1860 | 537 | 376 |  |
| Normal | - | Ref. | 0.821 (0.664, 1.016) | 1.280 (1.035, 1.583)* | 0.383 |
| Accelerated | - | Ref. | 1.017 (0.807, 1.282) | 1.511 (1.217, 1.877)* | 0.017 |

Multinomial logistic regression models were applied to evaluate the associations between SB consumption and biological aging status. Odds ratios (ORs) and 95% confidence intervals (CIs) are presented. The mitigated aging group was used as the reference category. Models were adjusted for age, sex, education level, physical activity, smoking status, drinking status, tea and coffee consumption, dietary energy intake, cardiovascular disease, diabetes mellitus, and kidney disease. **P* < 0.05.

**Supplementary Table 5.** Sensitivity analysis of the associations between sweetened beverage (SB) consumption and biological aging status, using the normal aging group as the reference category

|  | Non-consumer | Rare  (<1 serving/week) | Occasional  (1-2 servings/week) | Frequent  (≥3 servings/week) | *P*-trend |
| --- | --- | --- | --- | --- | --- |
| **All participants (n = 9104)** | 6054 | 2067 | 576 | 407 |  |
| Mitigated | Ref. | 0.869 (0.773, 0.978)* | 1.022 (0.903, 1.158) | 0.686 (0.606, 0.776)* | 0.014 |
| Accelerated | Ref. | 0.934 (0.824, 1.058) | 1.178 (1.053, 1.317)* | 1.168 (1.013, 1.345)* | 0.214 |
|  |  |  |  |  |  |
| **SB consumers (n = 3050)** | - | 2067 | 576 | 407 |  |
| Mitigated | - | Ref. | 1.189 (0.969, 1.459) | 0.780 (0.626, 0.971)* | 0.330 |
| Accelerated | - | Ref. | 1.289 (1.035, 1.606)* | 1.267 (1.024, 1.568)* | 0.021 |

Multinomial logistic regression models were applied to evaluate the associations between SB consumption and biological aging status. Odds ratios (ORs) and 95% confidence intervals (CIs) are presented. The normal aging group was used as the reference category. Models were adjusted for age, sex, education level, physical activity, smoking status, drinking status, tea and coffee consumption, dietary energy intake, cardiovascular disease, diabetes mellitus, and kidney disease. **P* < 0.05.

**Supplementary Table 6.** Associations between sweetened beverage (SB) consumption and biomarkers included in the biological age model

| Outcome | Non-consumer |  | Rare  (<1 serving/week) | *P*-value |  | Occasional  (1-2 servings/week) | *P*-value |  | Frequent  (≥3 servings/week) | *P*-value |
| --- | --- | --- | --- | --- | --- | --- | --- | --- | --- | --- |
| ALP | Ref. |  | -0.197 (-1.319, 0.926) | 0.731 |  | 1.996 (0.114, 3.878) | **0.038** |  | 3.893 (1.674, 6.112) | **<0.001** |
| ALB | Ref. |  | -0.001 (-0.139, 0.138) | 0.991 |  | -0.104 (-0.337, 0.128) | 0.379 |  | -0.052 (-0.326, 0.221) | 0.708 |
| BUN | Ref. |  | 0.059 (-0.010, 0.127) | 0.093 |  | 0.101 (-0.014, 0.216) | 0.085 |  | 0.159 (0.024, 0.295) | **0.021** |
| CR | Ref. |  | 0.542 (-0.284, 1.368) | 0.198 |  | 0.917 (-0.468, 2.303) | 0.194 |  | 0.567 (-1.066, 2.200) | 0.496 |
| SBP | Ref. |  | -0.291 (-1.104, 0.522) | 0.483 |  | 0.245 (-1.119, 1.609) | 0.725 |  | 0.840 (-0.766, 2.446) | 0.305 |
| FEV1 | Ref. |  | -0.013 (-0.038, 0.011) | 0.282 |  | 0.011 (-0.030, 0.052) | 0.591 |  | -0.019 (-0.067, 0.029) | 0.435 |
| FBG | Ref. |  | -0.044 (-0.091, 0.003) | 0.066 |  | -0.056 (-0.135, 0.023) | 0.166 |  | 0.020 (-0.074, 0.113) | 0.680 |
| BMI | Ref. |  | -0.011 (-0.184, 0.162) | 0.900 |  | 0.098 (-0.191, 0.387) | 0.506 |  | 0.699 (0.358, 1.040) | **<0.001** |
| GGT | Ref. |  | -1.533 (-3.190, 0.125) | 0.070 |  | -3.341 (-6.120, -0.562) | **0.018** |  | -2.594 (-5.870, 0.682) | 0.121 |
| PLT | Ref. |  | 1.992 (-1.193, 5.178) | 0.220 |  | 1.689 (-3.654, 7.031) | 0.536 |  | 6.103 (-0.200, 12.407) | 0.058 |
| WBC | Ref. |  | 0.070 (-0.011, 0.150) | 0.089 |  | 0.216 (0.081, 0.351) | **0.002** |  | 0.365 (0.206, 0.525) | **<0.001** |
| GOT | Ref. |  | -0.305 (-0.899, 0.289) | 0.314 |  | -0.270 (-1.266, 0.727) | 0.596 |  | -0.137 (-1.312, 1.037) | 0.819 |
| LDH | Ref. |  | 0.021 (-1.795, 1.837) | 0.982 |  | 3.627 (0.582, 6.672) | **0.020** |  | 10.258 (6.669, 13.847) | **<0.001** |

Multivariable linear regression models: Coefficients (95% conﬁdence interval). Models were adjusted for age, sex, education level, physical activity, smoking status, drinking status, tea and coffee consumption, dietary energy intake, cardiovascular disease, diabetes mellitus, and kidney disease.

Abbreviations: ALP, alkaline phosphatase; ALB, albumin; BUN, blood urea nitrogen; CR, creatinine; SBP, systolic blood pressure; FEV1, forced expiratory volume in one second; FBG, fasting blood glucose; BMI, body mass index; GGT, gamma-glutamyl transpeptidase; PLT, platelet count; WBC, white blood cell count; GOT, glutamic oxaloacetic transaminase; LDH, lactate dehydrogenase.

**Supplementary Figure 1.** Correlation matrix of biomarkers included in the biological age model

Abbreviations: ALP, alkaline phosphatase; ALB, albumin; BUN, blood urea nitrogen; CR, creatinine; SBP, systolic blood pressure; FEV1, forced expiratory volume in one second; FBG, fasting blood glucose; BMI, body mass index; GGT, gamma-glutamyl transpeptidase; PLT, platelet count; WBC, white blood cell count; GOT, glutamic oxaloacetic transaminase; LDH, lactate dehydrogenase.

**Supplementary Figure 2.** Associations between gut microbial α-diversity indices and biological age acceleration (BAacc)

Models were adjusted for age, sex, education level, physical activity, smoking status, drinking status, tea and coffee consumption, dietary energy intake, cardiovascular disease, diabetes mellitus, kidney disease and antibiotic use.

**Supplementary Figure 3.** Associations between five overlapping gut microbial genera and biomarkers included in the biological age model

The heatmap shows the associations between five overlapping gut microbial genera and biomarkers included in the biological age model. Colors represent standardized β coefficients from multivariable linear regression models, with red indicating positive associations and blue indicating inverse associations. Models were adjusted for age, sex, education level, physical activity, smoking status, drinking status, tea and coffee consumption, dietary energy intake, cardiovascular disease, diabetes mellitus, kidney disease and antibiotic use. **P* < 0.05, ***P* < 0.01, ****P* < 0.001.

Abbreviations: ALP, alkaline phosphatase; ALB, albumin; BUN, blood urea nitrogen; CR, creatinine; SBP, systolic blood pressure; FEV1, forced expiratory volume in one second; FBG, fasting blood glucose; BMI, body mass index; GGT, gamma-glutamyl transpeptidase; PLT, platelet count; WBC, white blood cell count; GOT, glutamic oxaloacetic transaminase; LDH, lactate dehydrogenase.
